# Empirical analysis on the impact of trust in government, purposes of GM crops, and farmers’ expectations on the delayed commercialization of GM crops in China———Based on Xinjiang and Guangdong survey samples

**DOI:** 10.1080/21645698.2023.2208996

**Published:** 2023-05-09

**Authors:** Yu Pang

**Affiliations:** Department of Management of Bioeconomy, Innovation & Governance, School of Social and Political Science, University of Edinburgh, Edinburgh, UK

**Keywords:** China, Delayed commercialization of GM crops, Farmers’ expectations, Government trust, Purposes of GM crops

## Abstract

GM crops, as a widely researched and applied biotechnology, hold significant strategic and practical implications for promoting the commercialization of GM crops in China, driving agricultural industry upgrading, and fostering economic and social development. However, despite their potential benefits, the commercialization of GM crops in China has been consistently delayed. Therefore, this study aims to explore the trust relationship between the government and the public in the field of genetically modified organisms and the heterogeneous impacts generated at the production and consumption ends. We primarily focus on insect-resistant cotton and genetically modified papaya as representatives and base our research on survey data from Xinjiang and Guangdong. By employing factor analysis and establishing multiple Probit models with government trust, crop purposes, and farmer expectations as independent variables, and the commercialization of GM crops as the dependent variable, we conduct two sets of empirical analyses. The study finds that government trust has a more significant impact on consumer concerns about whether to consume GM products than on producers, who prioritize farmers’ desire for agricultural product profitability. Age and education levels also influence the public’s acceptance of planting GM crops, but the effects are not as significant as the core variables mentioned earlier. Based on this, we conclude that consumers and farmers have different positions and contradictions in the specific scenario of delayed GM commercialization in China. In this context, this paper argues that diversified strategies should be adopted to address the issue of commercialization of GM crops in China.

## 1 Introduction

Over the past several years, genetically modified (GM) crops have been a widely debated topic, with their commercialization in the agricultural sector continually increasing.^1^ Driven by scientific research and global markets, an increasing number of countries have begun to accept and adopt this emerging agricultural technology. For instance, China has become the world’s largest importer of GM crops from countries such as the United States.^2^ Genetic modification endows crops with insect resistance and tolerance to certain herbicides, facilitating more efficient and sustainable food production. These are the two primary features of commercialization, allowing crops to yield higher outputs while reducing pressures from pests and weeds.^3^ As a result, GM crops have rapidly proliferated in the global agricultural landscape, with their development being substantiated by the agricultural conditions of numerous countries. The United States first approved the commercialization of GM tomatoes in 1996, after which the cultivation area for GM crops expanded rapidly worldwide. The United States, Brazil, Argentina, and Canada are currently the largest cultivators of GM crops, followed by India, which ranks fifth in adoption and cultivation with approximately 11.4 million hectares. China and the Philippines cultivate GM crops on 2.8 million and 0.6 million hectares, respectively.^4^ By 2019, 26 countries had adopted genetically modified biotechnology in the production of crops such as maize, soybeans, rapeseed, and cotton,^5^ yielding substantial environmental, health, economic, and social benefits.Although China’s GM industry has developed, it has faced several challenges. In the late 1980s, China began incorporating GM crops into its national development priorities to address food and cotton pest issues. The industry gradually developed and improved after 2000, and to promote GM technology research and development, the Chinese government significantly skewed fiscal expenditures toward it, investing nearly $4 billion between 2008 and 2020.^6^ To date, China’s commercial applications primarily involve GM insect-resistant cotton and GM papaya, cultivated since 1997 and 2006, respectively.^7^ Since 1997, China has conducted research on GM cotton technology and began commercial cultivation in 2000. Xinjiang, one of China’s representative regions for GM cotton commercialization, accounts for nearly 70% of the country’s total cotton cultivation area, making it one of China’s and the world’s most crucial cotton-producing regions. Guangdong, one of China’s essential papaya-producing areas, has been researching and commercializing GM papaya technology since 2006.^8^ In addition to domestic GM crop production, China’s import structure also includes overseas GM products. China’s GM soybean imports are the largest globally, accounting for over 60% of the country’s total soybean imports.^9^ Chinese farmers were among the first to cultivate GM crops,^10^ seemingly laying a solid foundation for the future development of GM crops in China. However, China’s GM crop industry has experienced a noticeable decline since 2010.^10^ For example, in 2012, policymakers chose to strengthen the regulation of GM crop research and commercialization to balance objectives such as promoting agricultural technology innovation, improving production efficiency, and ensuring national food security with concerns for ecological environmental protection and public confidence in GM crop safety. Consequently, the Chinese government suspended the commercialization of GM rice.^4^ The commercialization process of GM crops in China has been influenced by various factors. Among these, enhanced government regulation and scrutiny is a key factor. In 2016, the Chinese government released new regulations for GM crop approvals, strengthening oversight and review in this field,^11^ which has undoubtedly limited the commercialization of GM crops in China. Data from China’s National Bureau of Statistics shows that China’s GM crop cultivation area was 2.8 million hectares in 2019, a decrease of 0.9 million hectares, or 24.3%, compared to 2018.^12^ This indicates that the government’s scrutiny and regulation of GM crops have had a significant impact on the industry. Additionally, the delay in commercialization of China’s GM crop industry is related to social discourse and consumer attitudes. Many Chinese consumers are cautious about GM foods, concerned about their potential effects on human health and the ecological environment. This consumer mind-set also affects the promotion of GM crops and foods in the Chinese market.^2^ Furthermore, there are issues with research investment and technological innovation. Despite the Chinese government’s substantial funding in the GM field, China still lags behind international advanced levels in GM crop research and technological innovation.^4^ These factors collectively contribute to one of the most critical problems faced by China’s GM industry: the delay in commercialization.

With the introduction of China’s “14th Five-Year Plan for Bio-economy Development”^a^^a^China’s “14th Five-Year Plan for Bioeconomy Development” is an important economic development plan formulated by China for the period from 2021 to 2025, aiming to develop the biotechnology industry, improve the utilization efficiency of biological resources, and promote the sustainable development of the bio-industry. The plan covers various fields such as biopharmaceuticals, biomanufacturing, bio-agriculture, and bio-energy. in 2022, the Chinese government’s attitude toward the GM industry has entered a new phase of redevelopment. The development of the GM crop industry toward commercialization has always been an essential research issue that China must inevitably face. However, we must acknowledge that the plan is merely a blueprint for the future, and the more challenging and opportunistic the blueprint, the more necessary it is to clarify the past delay in China’s GM industry commercialization in a more transparent manner and structure. The delayed commercialization of GM crops in China is not entirely a spontaneous public behavior but a phenomenon closely related to government action.^13^ Therefore, the emergence of delayed commercialization of GM crops in China is a result of the combined influence of social public, farmers, and government policies. The interaction and feedback relationship between trust in the government and government actions has long been a focus of study in public administration. Previous literature has found that trust in the government is a critical factor in the commercialization process of GM crops.^14^ At the same time, the public will have different perceptions and acceptance levels for GM crops with different purposes, such as food crops or everyday economic crops, which in turn will affect the public’s consumption attitude toward GM crops. Another point worth noting is that with the continuous development of GM crop technology in China, it is not enough to rely solely on imports from other countries. Therefore, it is necessary to pay attention to the domestic farming community’s expectations for GM crops. Having positive expectations directly affects their planting preferences and, consequently, the production of GM crops.

This paper aims to start from the actual cognition and demand of Chinese public toward GM crops and use the actual questionnaire survey data on trust in the government, the purpose of GM crops, and farmers’ expectations from the delayed commercialization of GM crops in China. We seek to empirically understand the macro role of trust in the government and the impact of market consumption and supply on the commercialization of GM crops in China, taking into account the interests and concerns of consumers, farmers, and policy-makers to ensure the future safety and sustainable development of China’s GM crops. First, this paper reviews and comments on previous literature, collects first-hand survey data from community residents, farmers, and agricultural technology promoters in Guangdong and Xinjiang between 2021 and 2022, and establishes three fixed-effect Probit models to conduct targeted research on two sets of data from the same questionnaire. From a more systematic and market-oriented perspective of public and farmers’ consumption and supply, we explore the intrinsic relationship between trust in the government, the purpose of GM crops, and farmers’ expectations and the delayed commercialization of GM crops in China. Subsequently, based on the regression results, we address the issue of delayed commercialization of GM crops in China and provide some reasonable suggestions for the future commercialization development of China’s GM industry based on this foundation.

## 2 Literature Review and Theoretical Analysis

### 2.1 Trust in Government

Researchers worldwide pay close attention to the commercialization-related issues of genetically modified organisms, with relevant studies from the fields of communication, sociology, economics, political science, biology, and more.^7^ From the perspective of social public opinion and policy impact, there is a universal debate that the public initially lacks confidence in GM crops. However, as GM technology continues to develop and GM crops become commercialized, the public must face the potential threat signals of GM crops occupying their lives.^15^ This contradiction has, to some extent, affected the position of mass media and can be understood as a policy preference of the public. Therefore, governments in various countries must consider public opinion when formulating relevant policies.

This involves the issue of trust in government, which plays a crucial role in promoting socio-economic development, improving governance efficiency, and alleviating social contradictions. Sumran et al.,^4^ pointed out that Chinese consumers are unwilling to consider national institutions as sources of information, preferring self-generated opinions and rumors on social media. Moreover, the application of high technology in the food sector is not popular, reflecting people’s lack of trust in government supervision and technological safety. Lane^16^ divides trust in government into three aspects: cognition, institutions, and society. This paper mainly deals with the public cognition issues faced by the GM problem, which is closely related to the first dimension. Hu et al.^17^believe that in the GM field, the public often develops cognition based on the reliability of the government’s past related policies, thus making their judgments on GM products. Mayer et al.^18^ analyzed the dimensions of trust in government, including ability, benevolence, and integrity. Ability refers to the government’s capacity to provide administrative services; benevolence indicates that the government’s decision-making prioritizes the public’s interests, and integrity means that decision-making procedures adhere to the norms of legal decision-making, ensuring fair information disclosure processes. In the commercialization process of GM crops, there is a close relationship with these three dimensions. First, GM technology requires an efficient and capable administrative system to maintain related technology research and development and relevant market services, deploying the GM industry’s commercialization process nationwide. Second, it ensures the interests of GM crop farmers and the food safety of GM crop consumers. The public and related farmers are also very concerned about whether the involvement of other interest groups in the formulation of relevant policies will lead to unfair events, such as food accidents or impacts on the existing agricultural profit structure.^19^ Third, it is necessary to release relevant information and knowledge to the public in a timely manner to ensure their full participation in the GM commercialization development process.^20^

### 2.2 Different Purposes of GM Crops

In studying GM crop-related issues, it is evident that many factors influence public preferences.^21^ This indicates that GM crops, as a complex topic, have their public acceptance determined by various factors. Previous literature mainly focused on the public’s overall attitude toward GM crops without considering the differences among various GM crops. For instance, Cui and Shoemaker^15^ found that Chinese public attitudes toward GM crops were complex and contradictory, with both supportive and opposing voices. Knight and Paradkar,^22^ discovered that the Indian public held relatively negative attitudes toward GM crops, considering them as potential threats to health and the environment. In fact, from the fundamental perspective of influencing public attitudes, the differences in GM crops mainly lie in their application purposes in people’s daily lives. For example, GM crops as food sources (such as rice, flour, and papaya) and those used for daily life necessities (such as cotton) have different impacts on public perception and acceptance.^23^ Muringai et al.^24^ found that incorporating toxin genes into GM potatoes significantly reduced public acceptance of the crop. Rzymski and Królczyk^25^ found that public attitudes toward GM crops used for pharmaceutical production were relatively positive, as these crops typically do not enter the food chain directly and possess evident medical value. Once GM crops are involved in the human body’s energy cycle, they will have tangible effects on human health, influencing people’s attitudes and preferences toward GM crops. In contrast, GM products used in daily life are often cheaper, and the public may overlook some GM-related information for the sake of convenience.

Exploring the differences in public attitudes and acceptance toward different types of GM crops and their application fields, rather than merely studying the overall attitude toward GM crops, is crucial for China’s GM commercialization research. This will help formulate more purposeful GM-related policies. The Chinese government has different attitudes toward various GM crops, currently granting safety certificates only for some GM products such as GM cotton and GM papaya. Papaya and cotton also serve as the basis for differentiation based on edibility, providing conditions for the related research in this article. In 1997, China approved the commercial cultivation of GM cotton, followed by the approval of GM papaya commercial cultivation in 2006.^4^ Since then, China has not approved the commercial cultivation of other similar GM crops. Although some varieties have made progress in the safety approval process, they have not yet received commercialization approval,^7^ such as GM corn and GM rice, hindered by potential safety risks. GM corn might produce toxic effects on non-target insects, and although it has passed safety approval, it has not received commercialization approval.^26^ The commercialization process of GM rice has been delayed for nearly 20 years.^27,28^ Among the approved GM products, the required time for approval varies, reflecting the government and public concerns about the safety and ecological impact of edible crops. Paying attention to these differences also deepens the exploration of the delayed commercialization of China’s GM industry.^29^

### 2.3 Farmers’ Expectations

Farmers are the primary cultivators of GM crops and the starting point of GM commercialization structure. Their views on whether GM crops can promote sustainable food production and their outlook on the future profitability of GM crops are critical to understanding the delay in China’s GM commercialization. Their expectations and preferences are among the essential factors influencing the commercialization process of GM crops in China.^4^ Studies have shown that farmers’ acceptance and willingness to use GM crops, their perception and evaluation of potential benefits and risks, their social networks, and information channels might affect their attitudes and preferences toward GM crops.^30^ For instance, Sumran et al.,^2^ posits that farmers’ acceptance of GM crops is closely related to their views on the economic benefits, environmental impact, and safety of such crops. Additionally, they mentioned the impact of psychological distance on farmers’ adoption of GM crops. Moreover, government policies and regulations, media coverage of GM crops, and public education could also influence farmers’ expectations and preferences.^31^ Guo et al.^32^ investigated the attitudes and expectations of 500 farmers from 13 districts in Henan Province, China, toward GM corn through questionnaires and field interviews. The study found that most farmers supported GM corn, but there were also concerns and mistrust to some extent. Other research has indicated that farmers’ expectations depend on their experience in cultivating GM crops,^33^ and the sustainability of the existing GM cultivation system is sufficient to prove and motivate farmers to have greater demand for expanding production.^34^ This can be understood as the profitability and availability of GM crops helping farmers strive to achieve new professional goals. Overall, if farmers have positive expectations for GM crops, it would undoubtedly provide fundamental benefits for the development of GM crops on the production supply side. Such positive expectations originate from the farmers’ thoughts, and their influence is arguably more direct and thorough than the impact of government policies.^35^ However, if GM technology does not have a significant advantage in farmers’ expectations, it may also hinder the commercialization of GM crops in China. This forms a push-pull mechanism on the level of farmers’ expectations for GM crop commercialization, with GM crops influencing farmers’ expectations, and vice versa, farmers’ expectations also affecting the production of GM crops.

### 2.4 Critical Analysis

With the growing population and increasing scarcity of resources, genetic modification technology, as a new biotechnology, has attracted widespread attention and research globally. While some have studied the drawbacks and risks of large-scale cultivation of GM crops, such as increased pest resistance and gene contamination,^36^ others argue that goals like pest control and yield increase, which are expected from GM crops, can also be achieved through alternative methods such as ecological cycles, new variety development, hybridization, and breeding improvement, without facing the risks associated with GM crops.^37^ However, despite these studies, the total global cultivation area of GM crops continues to increase,^5^ reflecting a bright developmental outlook for GM crops.

In China, however, the commercialization process of GM crops has encountered some obstacles. To date, China has gone through a series of safety approval processes, but only a few varieties have been approved for commercialization, while most GM crops remain under review. China’s GM commercialization is primarily supported and driven by the government,^38^ so past research has focused mainly on the impact of decision-making systems or authoritative figures on GM commercialization. Scholars like Xiao and Kerr^7^ analyzed this phenomenon from a political economy perspective and by comparing foreign GM industry regulatory policies, while Cui and Shoemaker^15^ studied consumers’ overall attitudes toward GM crops. However, these studies are based on government decision-makers or consumer perspectives.^39^ Even when public opinion is considered, it originates from the views of authoritative institutions or figures or the influence of public media platforms on policy changes.^40^ These studies do not adequately consider the relationship between public attitudes and government decisions, nor do they take into account the differences in public preferences and roles in the GM industry, such as producers and consumers. These detailed changes have led to a dynamic compromise between the Chinese government and the public on GM crops, which is a crucial factor influencing past commercialization delays. Previous research has also focused on consumers’ or farmers’ psychological distance and attitudes toward GM crops individually without considering the different purposes of various GM crops and their direct effects on government trust and farmers’ expectations or connecting consumption and production sides in the same dimension.^2,4^ To be more specific, the public and farmers represent the primary consumers and producers in China’s commercialization of genetically modified crops. By investigating the actual preferences and expectations of the Chinese public and farmers toward genetically modified crop products from both consumption and production perspectives, we can explore their trust in the government and the heterogeneous impact of the purposes of genetically modified crops on the commercialization at the consumption and production levels. There is a lack of comprehensive research on the delayed commercialization of genetically modified crops in China from the perspectives of consumption, production, the crops themselves, and the government. Therefore, analyzing this phenomenon from a perspective that has been missing in previous literature on the delay in China’s commercialization of genetically modified crops is the primary issue this article aims to address.

## 3 Empirical Analysis of Questionnaire Survey

### 3.1 Data Processing and Model Setting

To date, China has primarily commercialized the cultivation of Bt transgenic cotton (since 1997) and transgenic papaya (since 2006).^4^ Xinjiang, situated in the northwest of China, is the nation’s largest transgenic cotton-growing region, while Guangdong, located along the southeastern coast, is a significant transgenic papaya production area. The substantial geographical disparities between these regions provide an excellent opportunity to explore the overall attitudes and acceptance levels of consumers and farmers toward GM crops across different areas in China. Furthermore, these two GM crops represent both food and cash crops, and examining them can offer valuable insights into the application and promotion of GM technology across various agricultural sectors, ultimately contributing to a comprehensive understanding of the actual cultivation of these GM crops in China. As a result, this study focuses on Guangdong and Xinjiang as representative research areas for the collection of primary survey data, with investigators conducting on-site questionnaire surveys across both regions. Specifically, the study utilized face-to-face interviews, telephone interviews, and online surveys to distribute questionnaires to local residents. In face-to-face interviews, researchers visited farms, markets, and communities to engage with respondents; during telephone interviews, researchers contacted randomly selected residents from various regions; and online surveys were conducted via social media, e-mail, and online survey platforms. Such a diversified approach aids in ensuring the validity and reliability of the survey results.

The survey questionnaire consists of 21 questions, divided into 5 main sections, aiming to comprehensively and in-depth assess the respondents’ knowledge, attitudes, and actual behaviors toward GM crops while also focusing on the expectations and needs of farmers in the commercialization process of GM crops. The sections are as follows: First, basic information collection, which primarily gathers background information on the respondents, such as age, gender, education level, and occupation, in order to analyze the relationship between the public’s attitude toward GM crops and their socio-economic background. Second, understanding of GM crops, where a series of questions assess the respondents’ knowledge of the definition, principles, and safety aspects of GM crops, as well as their sources of related information. Third, attitudes toward GM crops, which explores respondents’ views on the safety, environmental impact, and economic benefits of GM crops, as well as their preferences for government policies in promotion and regulation. Fourth, purchasing and using GM products, focusing on respondents’ actual experiences of buying and using GM products in daily life, such as purchase frequency, primary considerations, and willingness to pay a premium for non-GM products, revealing the respondents’ acceptance of GM products during consumption. Fifth, the expectations and needs of farmers, specifically targeting farmer respondents, delving into their demands and expectations regarding GM crop cultivation, such as concerns about cultivation technology training, policy support, and market information. Through a detailed analysis and discussion of this data, we hope to reveal the roles and positions of different groups in the commercialization process of GM crops. This paper provides an appendix with detailed information about the survey questionnaire at the end of the article.

Apart from adding questions about farmers’ expectations due to their identity in the final section, there are no differences in other parts of the questionnaire. This helps maintain consistency in the main variables and, in the process of separately analyzing farmers and other public respondents in regression analysis, prevents any judgment biases from affecting the results due to the influence of other potential variables on consumers and producers.

Due to restrictions such as epidemic prevention and control measures, the questionnaire survey spanned from January 2021 to July 2022. A total of 457 questionnaires were collected, with 433 valid questionnaires, yielding an effective rate of 94.7%. Among the valid questionnaires, 255 were from Xinjiang and 178 from Guangdong. In 2021, 310 questionnaires were obtained, with 172 from Xinjiang and 138 from Guangdong; in 2022, 123 were obtained, with 83 from Xinjiang and 40 from Guangdong. The survey questionnaires essentially covered all regions of Xinjiang and Guangdong.

As this study starts from the actual expectations of the public, it is crucial to distinguish the different roles of various public groups in the commercialization of GM crops. From the perspectives of demand and supply, they can be divided into general consumers and farmer producers. Among all valid questionnaires, 82 were filled out by farmers in Xinjiang and 56 by farmers in Guangdong, making a total of 138 questionnaires filled out by farmers. The remaining 295 valid questionnaires were filled out by consumers. Based on these two research subjects, the data is divided into two groups. The first group consists of panel data from 2021 to 2022 for Xinjiang and Guangdong, excluding farmers. The second group comprises panel data filled out by farmers in Xinjiang and Guangdong from 2021 to 2022.

The Probit model is a classical generalized linear model, first proposed and applied by Chester Bliss in the 1930s.^41^ This analytical method has been widely used in social science research to evaluate public intentions, such as Huanguang^42^ using the Probit model to analyze consumers’ willingness to choose in a study on the relationship between government trust and consumers in relation to GM crops. The reason for choosing the Probit model in studying the delayed commercialization of GM crops in China is that it is suitable for dealing with latent continuous variables and is relatively robust when handling extreme data points. By defining dependent and explanatory variables and using the maximum likelihood estimation method to estimate model parameters, the Probit model can be employed to analyze the extent and relationships of various factors on the delayed commercialization of GM crops. The study aims to explore the significance of government trust, GM crop purposes, and farmers’ expectations, evaluating the presence and extent of these factors’ influences.

This study plans to establish three probit models for the two groups of data.

First, we set the variables for Model 1 of these two panel data groups.

The two baseline regression equations are:

(1) $$Y_{1}=a_{0}+a_{1}Trust+a_{2}Purpose+\varepsilon$$

(2) $$Y_{2}=a_{0}+a_{1}Expectation+\varepsilon$$

Y1 represents whether the consumer public is willing to purchase GM products, and Y2 represents whether farmers are willing to plant GM products; α0 represents the model constant term; Trust represents the trust in government factor; Purpose represents the purpose factor of GM crops; Expectation represents the expectation factor of farmers; ε represents the error term. α1 and α2 are the coefficients of the two regression equations.

Next is the setting of variables for Model 2 of these two panel data groups. The two baseline regression equations are:

(3) $$Y_{1}=a_{0}+a_{1}Trust+a_{2}Purpose+a_{3}Controls+\varepsilon$$

(4) $$Y_{2}=a_{0}+a_{1}Expectation+a_{2}Trust+a_{3}Purpose+\;a_{4}Controls+\varepsilon$$

In both data groups, Controls are introduced simultaneously. Controls represent three control variables, which are age, gender, and education level. The other variables have been explained earlier and will not be elaborated on here.

Finally, the setting of variables for Model 3 of these two panel data groups is as follows. The two baseline regression equations are:

(5) $$Y_{1}=a_{0}+a_{1}Trust+a_{2}Purpose+a_{3}Trust*Purpose+a_{4}Controls+\varepsilon$$

(6) $$Y_{2}=a_{0}+a_{1}Expectation+a_{2}Trust+a_{3}Purpose+\;a_{4}Expectation*Trust+a_{5}Expectation*Trust+a_{6}Controls+\varepsilon$$

To further investigate the interaction and influence among explanatory variables, as well as the impact of these interactions on the dependent variables, cross-variables are introduced. Since all the mentioned explanatory variables are binary dummy variables, each interaction term will generate four interaction scenarios. In the consumer group, the cross-variable Trust*Purpose is introduced; in the producer group, the cross-variables Expectation*Trust and Expectation*Purpose are introduced.

Since the data in this paper are panel data, it is necessary to test whether a fixed-effects model or a random-effects model should be used for regression analysis. The results of the Hausman test indicate that Model 1, Model 2, and Model 3 should all use the fixed-effects model. (Tables 3 and 5)

**Table 1.** Variable Definitions.

	Variable Name	Variable Definition	Measurement Method
Dependent Variable	Purchase	Consumer Purchase Intention	Consumers’ willingness to purchase GM (GM) crop products: 1 = willing to buy; 0 = unwilling to buy.
	cultivation	Farmers’ Cultivation Intention	Farmers’ willingness to cultivate GM products: 1 = willing to cultivate; 0 = unwilling to cultivate.
Explanatory Variable	Trust	Trust in Government	Whether the respondents feel that the government timely releases relevant GM information and disseminates knowledge: 1 = yes; 0 = no
	purpose	Purpose of GM Crops	Whether respondents care if GM products are used for consumption or daily use: 1 = yes; 0 = no.
	Expectation	Farmers’ Expectations	Whether cultivating GM crops can improve their income in the future: 1 = yes; 0 = no.
Control Variable	Gender	Gender	Respondents’ gender: 1 = male; 0 = female.
	Age	Age	1 = 18–25 years old; 2 = 26–39 years old; 3 = 39–60 years old; 4 = 61 years old and above.
	Education	Education Level	1 = elementary school and below; 2 = junior high school; 3 = high school; 4 = college; 5 = university and above.
Interaction Variable	Trust*Purpose	Trust in Government * Purpose of GM Crops	Interaction between trust in government and the purpose of GM crops.
	Expectation*trust	Farmers’ Expectations * Trust in Government	Interaction between farmers’ expectations and trust in government.
	Expectation*purpose	Interaction between farmers’ expectations and government trust.	Interaction between farmers’ expectations and the purpose of GM crops.

**Table 2.** Descriptive Statistics of Variables for Consumer Group Data and Producer Group Data.

Consumer Group						roducer Group					
Variable	Sample Size	Mean.	Standard Deviation	Min	Max	Variable	Sample Size	Mean.	Standard Deviation	Min	Max
Purchase	295	0.7027	0.4578	0	1	cultivation	138	0.8333	0.3740	0	1
Trust	295	0.5878	0.4931	0	1	Trust	138	0.7536	0.4324	0	1
purpose	295	0.4189	0.4942	0	1	purpose	138	0.4565	0.4999	0	1
Gender	295	0.6149	0.4874	0	1	Expectation	138	0.7536	0.4324	0	1
Age	295	34.4628	12.8719	18	66	Gender	138	0.5942	0.4928	0	1
Education	295	3.3986	1.2198	1	5	Age	138	36.0144	14.6062	18	71
Trust*Purpose	295	0.3581	1.48026	0	1	Education	138	3.4928	1.2743	1	5
						Expectation*trust	138	0.6159	0.4881	0	1
						Expectation*purpose	138	0.3478	0.4780	0	1

**Table 3.** Fixed Effects Probit Model Estimation for Consumer Group.

	Model 1		Model 2		Model 3	
Explanatory variable	Dependent variable（Y）: Whether the public is willing to buy (Buy=1; Not buy=0)					
	Coefficient	Z-Statistic	Coefficient	Z-Statistic	Coefficient	Z-Statistic
Trust	1.890***	5.894	1.805***	5.401	1.650***	4.443
Purpose	1.396***	3.652	1.414***	3.582	1.740***	3.113
Gender			0.515	1.601	0.519	1.612
Age			−0.019	−1.562	−0.019	−1.611
Education			0.753**	1.984	0.769**	2.023
Trust*purpose					−0.681*	−0.874
Loglikelihood	−132.103		−132.197		−131.814	
R2/Pseudo R2	0.267		0.266		0.268	
Hausman test	96.26***		107.77***		120.63***	
Total obs	295					

Note. *p<.10, **p<.05, ***p<.01, software used: Stata.17.

### 3.2 Variable Setting and Descriptive Statistics

Dependent variables: The most intuitive reflection of the delayed commercialization of GM crops can be found in two sets of data: the willingness of consumers to purchase GM crops and the willingness of farmers to plant them. Therefore, the first set of data uses the willingness of consumers to purchase GM products as a proxy variable, with willing to purchase = 1 and unwilling to purchase = 0. For the perspective of farmers as suppliers and producers, their willingness to plant GM products is used as a proxy variable, with willing to plant = 1 and unwilling to plant = 0.

Explanatory variables: In the questionnaires, several concerns about GM crops were raised by the surveyed public (including consumers and producers). A total of nine factors were summarized, including the perception of an efficient and capable administrative system for GM crop commercialization, past policies that safeguard their interests and food safety, timely release of GM information and knowledge dissemination by the government, whether they care about GM products being used for consumption or daily use, whether they would continue to consume products containing GM ingredients if they had already purchased them, whether they prefer non-GM or GM products if GM crop products are cheaper, whether they have prior experience with planting GM crops, their outlook on GM crops improving agricultural productivity and soil conservation, and their expectations for income growth from planting GM crops.

A principal component analysis was conducted on these nine factors, extracting three principal component factors.^b^^b^Principal components were extracted based on a cumulative contribution rate>0.8, using SPSS27. Due to space limitations and the research focus of this article, the specific process of principal component analysis is not presented here.,^43^ According to the factor loading matrix, the first principal component had larger absolute loadings on “perception of an efficient and capable administrative system for GM crop commercialization,” “past policies that safeguard their interests and food safety,” and “timely release of GM information and knowledge dissemination by the government.” This represents the trust in government factor, which refers to the public’s overall trust in the government regarding GM food commercialization. Examples include perceptions of government protection, acceptance of government efforts in promoting GM food concepts (especially breaking through comfort zones due to trust in the government and unfamiliarity with the commercialization process), and acceptance of the uncertainty of GM crop safety.

The second principal component had larger absolute loadings on “whether they care about GM products being used for consumption or daily use,” “whether they would continue to consume products containing GM ingredients if they had already purchased them,” and “whether they prefer non-GM or GM products if GM crop products are cheaper.” This represents the purpose of GM crops factor, which refers to the public’s understanding of GM crop products from a purpose perspective when they are truly integrated into their lives.

The third principal component had larger absolute loadings on “whether they have prior experience with planting GM crops,” “their outlook on GM crops improving agricultural productivity and soil conservation,” and “their expectations for income growth from planting GM crops.” This represents the farmers’ expectation factor, which refers to the overall perception of GM crops by farmers.

Based on the results of this principal component analysis, the independent variables for the two sets of data are set as “trust in government,” “purpose of GM crops,” and “farmers” expectations.” In the econometric analysis process, the principles and methods of proxy variables in econometrics were used. “Trust in government” is replaced by its largest absolute loading variable, “whether the government timely releases relevant GM information and disseminates knowledge.” In the questionnaire, selecting “yes” is assigned a value of “1,” and all other values are assigned “0.” The “purpose of GM crops” is replaced by its largest absolute loading variable, “whether they care about GM products being used for consumption or daily use.” In the questionnaire, selecting this option is assigned a value of “1,” and all other values are assigned “0.” “Farmers” expectations” are replaced by its largest absolute loading variable, “whether planting GM crops can improve their income in the future.” In the questionnaire, selecting this option is assigned a value of “1,” and all other values are assigned “0.”

Control Variables: Since the basic questionnaire content is completely identical except for the unique responses from farmers, the control variables introduced in the second set of data can be the same. Three control variables are introduced in both sets of data, in addition to the core independent variables, to examine whether the significance between the core independent variables and the dependent variable changes under the influence of a series of control variables. The control variables are mainly designed to consider the individual characteristics of the public (i.e., the heterogeneity of each respondent). The individual characteristics of the public are represented by three variables: age, gender, and education level.

Interaction Variables: To further examine the interaction and influence among the independent variables and their impact on the dependent variable after interaction, interaction variables are introduced. Since all of the mentioned independent variables are binary dummy variables, each interaction term will generate four interaction situations. In the first set of data, the main interaction term is between “trust in government” and “purpose of GM crops.” In the second set of data, the main interaction terms are between “trust in government” and “farmers” expectations” and between “purpose of GM crops” and “farmers” expectations.” These independent variables still use proxy variables.

By organizing the questionnaire data, we set the variable definitions as shown in Table 1 and the descriptive statistics of the variables as shown in Table 2 for both datasets.

## 4. Regression Results and Analysis

### 4.1 Fixed-Effects Probit Model Estimation for the Consumer Group

In the three models, the two core explanatory variables selected in this study, “trust in government” and “purpose of GM crops,” are both significant at the 1% significance level. This indicates that “trust in government” and “purpose of GM crops” have a significant impact on the public’s consumption of GM crop products and are important factors affecting the delayed commercialization of GM crops in China. The significance coefficients of both trust in government and purpose of GM crops factors are positive, indicating that the more the public feels the government timely publishes relevant GM information and disseminates knowledge, the better their understanding of GM products will be, and the stronger their desire to buy GM crop products. In addition, this also shows that the public has a clear difference in attitudes toward GM crops for daily food consumption or daily life use. For daily food consumption, the public tends to be conservative toward GM crop products, while for daily necessities, if the price is appropriate, there is no significant aversion compared to ordinary products. Among the two explanatory variables, the Z value of “trust in government” is the largest, indicating that among the two factors selected, “trust in government” is the primary factor affecting the delayed commercialization of GM crops in China.

In Model 2, the influence of “education level” is less significant compared to “trust in government” and “purpose of GM crops,” and its impact level is limited. The significant coefficient of “education level” is positive, indicating that education has a certain impact on whether the public is willing to buy GM crop products. Generally, the higher the public’s education level, the higher their acceptance of GM products. Specifically, people with higher education levels usually possess stronger scientific literacy and technological awareness, allowing them to better understand and accept new technologies and developments. They are more likely to accept and comprehend the development trends and application scenarios of science and technology, have a deeper understanding and appreciation of the importance of technological innovation, and thus have a more open attitude and perception toward GM technology. People with lower education levels may lack scientific knowledge and technological literacy, making them more sensitive to the risks and uncertainties of GM technology and prone to worry and resistance. They often pay more attention to the originality and purity of food and may have misconceptions or misunderstandings about the concept and process of GM food, resulting in distrust and rejection.

Further analysis of the cross-term regression results in Model 3 reveals that the factors of “trust in government” and “purpose of GM crops” interact with each other. As we mentioned earlier, the advantage of interaction term regression lies in distinguishing four different types of public comprehensive opinions. To facilitate the interpretation of the interaction term regression coefficients, we transcribe the results of Model 3‘s interaction terms into Table 4. Table 4 shows that, in terms of the public’s willingness to purchase GM crop products, the willingness of those who feel trust in government factors and are indifferent to the purpose of GM crops factors is the highest, followed by those who feel trust in government factors and care about the purpose of GM crops factors, those who do not feel trust in government factors and are indifferent to the purpose of GM crops factors, and those who do not feel trust in government factors and care about the purpose of GM crops factors. This is consistent with the conclusions of our previous regression analysis, which shows that “trust in government” has a stronger influence on the public’s willingness to buy GM products, while “purpose of GM crops” also increases the public’s willingness to buy GM crop products. In China’s context, resolving the issue of trust in government is the most critical factor in delaying GM commercialization at the consumer level.

**Table 4.** Summary of Trust*Purpose interaction term regression coefficients.

Public category	Whether to buy
Trust=1\|purpose=1	0.794
Trust=1\purpose=0	0.953
Trust=0\purpose=0	0.667
Trust=0\purpose=1	0.394

The public, situated in the contradiction between government and market, consumption and production, are at a relative disadvantage because their interest demands cannot be directly expressed in the commercialization process of GM crops. At most, they can express their opinions indirectly through social media or consumer choices. Moreover, according to the regression results, the choices of the general public are often strongly influenced by the government and the purpose of GM crops. This means that they not only lack direct means to influence the commercialization of GM crops, but their remaining indirect means may also be insufficient for making rational choices based on their interests. Specifically for GM crops, the government and large state-owned GM enterprises in China cannot respond to public interests promptly, as their preferences focus on meeting the overall food abundance and technological development needs of the country. However, the public is more concerned with the purpose and safety of GM crops, which becomes a source of conflict in consumer attitudes. From the perspective of public interests and standpoint, consumers desire safe food but are influenced by the degree of trust in the government and the externalities of GM products. In this situation, the discrepancy in interests between the public and the government will inevitably lead rational consumers to be skeptical about GM crops. This skepticism is particularly strong in a vast country like China, where the national government and state-owned GM enterprises are more inclined to focus on overall food supply and technological progress. Ultimately, if China continues to maintain a delayed commercialization of GM crops, the skeptical attitudes of consumers will need an outlet, further becoming a new driving force hindering the commercialization of GM crops at the consumer level. This creates a vicious cycle of delayed commercialization of GM crops in China at the consumer level, intensifying the delay in commercialization of GM crops at the consumption end.

### 4.2 Fixed Effects Probit Model Estimation for the Production Group

In all three models, the core explanatory variable “expectations” is significant at the 1% level, indicating that “expectations” have a significant impact on whether farmers will plant GM crops and is an important factor affecting the delayed commercialization of GM crops in China. In Models 2 and 3, “trust” is significant at the 5% level. The significant coefficients of both farmers’ expectation factors and trust in the government are positive, indicating that the better the future income expectations for GM crops among farmers, the stronger their desire to plant GM crops. Farmers are more willing to plant GM crops when they feel that the government timely releases GM-related knowledge and information. “Purpose” is not significant, and whether farmers care about GM crop products for food or daily use has no significant impact on their willingness to plant GM crops. Among the significant explanatory variables, the Z-values of “expectations” are the largest, indicating that this variable has the strongest significance. This shows that among the three selected factors, “expectations” is the primary factor affecting the delayed commercialization of GM crops in China. Secondly, compared to the coefficients of the entire consumer group data, the coefficients of farmers are generally larger than those of the public, indicating that farmers are generally more sensitive to explanatory variables, and whether they are willing to plant GM crops is more affected by future income expectations and trust in the government. According to the regression results, the production group is generally more sensitive to explanatory variables than the consumption group, and farmers are more affected by trust in the government than the consumption field, which means that the government has greater potential for return on investment in the production end in the future.

In Model 2, the three individual characteristic control variables, “age” and “education level,” have relatively low significance compared to “expectations” and “trust” and have limited impact. The coefficient of “age” is significantly negative, while the coefficient of “education level” is significantly positive. This shows that both age and education level have an impact on farmers’ acceptance of planting GM crops. On the one hand, age is a factor affecting farmers’ acceptance of planting GM crops. Older farmers may not be as accepting of new technologies as younger farmers because they may have a lower understanding of new technologies and a deeper emotional identification with traditional agricultural methods. Younger farmers are more likely to accept new technologies because they are more open to new things and have access to more information. On the other hand, education level is also one of the factors affecting farmers’ acceptance of planting GM crops. Farmers with higher education levels are usually more receptive to new technologies and ideas, and they can better understand the advantages and safety of GM technology, making them more willing to accept and plant GM crops.

By further analyzing the cross-item regression results of Model 3 in the second set of data, we can find the influence of the two interaction items, “expectations” and “trust,” and “expectations” and “purpose.” The advantage of the interactive-item regression is that it distinguishes different types of farmers’ comprehensive views. The regression results show that the interaction item “expectations” and “trust” have a significant impact. To facilitate the interpretation of the coefficients of the interaction item regression, we transcribe the results of the interaction item “expectations” and “trust” of Model 3 into Table 6. Table 6 shows that, in terms of farmers’ willingness to plant GM crops, those who have a better future income expectation for GM crops and feel trust in the government have the highest willingness. Then, in order, are those who have a better future income expectation for GM crops but do not feel trust in the government, those who have a worse future income expectation for GM crops but feel trust in the government, and those who have a worse future income expectation for GM crops and do not feel trust in the government. Both “expectations” and “trust” have a strong impact on farmers’ willingness to plant GM crops and can increase their willingness to plant GM crops in the future, with the impact of “farmer expectations” being the most significant.

**Table 5.** Fixed Effects Probit Model Estimation for the Production Group.

	Model 1		Model 2		Model 3	
Explanatory variable	Dependent variable（Y): Whether the farmer is willing to plant (Plant=1; Not plant=0)					
	Coefficient	Z-Statistic	Coefficient	Z-Statistic	Coefficient	Z-Statistic
Expectation	5.241***	4.91	5.866***	3.72	4.229***	2.37
Trust			3.932***	2.53	3.624**	2.25
Purpose			−0.311	−0.29	0.170	0.14
Gender			−.0342	−0.03	0.264	0.22
Age			−0.106**	−2.74	−0.108	−2.71
Education			2.136 **	1.75	2.357**	1.80
Expectation*trust					−1.872***	−2.46
Expectation*purpose					0.614	0.37
Loglikelihood	−247.34		−288.96		−296.31	
R2/Pseudo R2	0.589		0.749		0.569	
Hausman test	143.86***		155.38***		162.33***	
Total obs	138					

Note. *p<.10, **p<.05, ***p<.01, software used: Stata.17.

**Table 6.** Summary of regression coefficients for the Expectation*trust interaction term.

Farmer Category	Planting Decision
Expection=1\trust=1	5.302
Expection=1\trust=0	4.762
Expection=0\trust=0	0
Expection=0\trust=1	1.977

Farmers are the direct implementers of government policies. According to the regression results, the farmers’ own interest expectations are the biggest influencing factor, and the trust in the government has a significant impact but is not the deciding factor. This actually has a deeper meaning, which is that even if the agricultural sector has full trust in the government in terms of GM crops, China’s planting and production of GM crops can still be easily affected by the external environment. As long as it meets the farmers’ positive expectations for the future, even if they cannot be sold domestically, farmers may be willing to take legal risks to plant GM crops and export them to foreign countries. From a probability perspective, the planting of GM crops by farmers is almost an inevitable event because farmers pursue economic benefits and expectations. Even if there is a lack of trust in the government regarding GM crops, farmers tend to plant GM products for the profit margin generated by the increased agricultural production efficiency brought by GM technology. Moreover, the Chinese government is increasingly showing support for GM products overall,^27^ providing support for farmers to plant GM crops. Trust in the government and farmers’ own expectations both affect the delayed commercialization of GM crops in China. Therefore, the current delay in the commercialization of GM crops in China at the production level implies that the previous delay in the commercialization of GM crops was actually the result of a compromise of interests at various levels. Farmers’ expectations have not been fully met, but with the advancement of global GM technology and the reduction of production costs, the external environment is constantly improving. Developing GM crops at the production level is almost an inevitable choice for China, which is actually a favorable boost for the government’s active development of GM crop commercialization.

### 4.3 Integrated Discussion of Consumer and Producer Groups

Comparing the significant influencing factors between the production and consumption groups horizontally, although trust in the government has a significant impact on both consumption and production, it is important to note that in the eyes of farmers, the impact of expectations for GM crops outweighs the impact of trust in the government. In the eyes of the general public as consumers, trust in the government has a more significant impact than the purpose of GM crops. The reason for this is that GM crops are essentially agricultural products, and the relationship between farmers and agriculture is a very close and dependent one. They rely on producing more profitable agricultural products to maintain production, and they have no production freedom beyond choosing profitable products. The lack of a significant correlation between the purpose of GM crops and whether farmers plant GM crops is also proof of this point. As for the general public as consumers, regardless of whether they are skeptical about GM crops, they still have the consumption freedom to purchase traditional non-GM crop products. The different purposes of GM crops and the different interaction methods in the production and consumption sectors are the essential differences and distinctions.

In terms of age and education level, there are similar impacts in both the consumption and production sectors. This indicates that this aspect plays a role in bottoming out the delayed commercialization of GM crops in China. Education level or education bias can also be a reason to explain the different overall attitudes toward GM crops among different national groups in China and the international community.

## 5. Conclusions and Recommendations

For decades, genetically modified (GM) crops have been a controversial topic. Since their commercialization in 1996, GM crops have been extensively cultivated and sold worldwide. However, in China, the commercialization of GM crops has been delayed due to various factors.^7^ Despite the many potential benefits of GM crops, there are still some uncertain risks. These risks raise concerns and opposition among the public and farmers, which in turn affect government decision-making and policies.^44^ As the needs of national strategic arrangements and social development continue to evolve, simply delaying or stagnating is not the solution. It is crucial to explore the relationship between consumers, producers, GM crops, and government policies from a perspective that closely aligns with the actual needs and demands of the public and farmers. Therefore, this study investigates the perception, needs, and expectations of the public and farmers toward GM crop products. It also examines the interaction between GM crops and the public and the influence of government policies on public perception. Two sets of data and three fixed-effects Probit models are established to analyze the impact of trust in the government, the purpose of GM crops, and farmers’ expectations on the delayed commercialization of GM crops in China. Compared with existing studies, this research specifically focuses on the key role of government trust among consumers and producers, as well as the different purposes of GM crops in the minds of consumers and farmers. Additionally, the study analyzes the impact of education levels on the acceptance of GM crops by the public and farmers, highlighting the importance of improving scientific literacy in promoting public acceptance of new technologies. These findings provide new perspectives and strategies for addressing the delayed commercialization of GM crops in China, facilitating the development and implementation of relevant policies. The study’s findings include

For consumers, factors such as trust in the government and the purpose of GM crops have a significant impact on the delayed commercialization of GM crops in China, acting as essential factors promoting or restraining this delay. Among these factors, trust in the government has the most significant impact on the delayed commercialization of GM crops. Furthermore, there is a considerable difference in consumers’ perception of GM crops depending on whether their primary use is for consumption or everyday use. For the general public, GM crops intended for consumption imply a higher threshold of trust and safety requirements. Finally, while education levels do affect the public’s acceptance of GM crops, the effect is not as significant as the core variables mentioned earlier. Individuals with higher education levels are more open and receptive to new technologies and innovations, while those with lower education levels tend to be more conservative and cautious.

For producers, factors such as farmers’ expectations and trust in the government significantly impact the delayed commercialization of GM crops in China, acting as essential factors promoting or restraining this delay. Among these factors, farmers’ expectations have the most significant impact on the delayed commercialization of GM crops. Farmers’ anticipation of increased future income from planting GM crops encourages them to accept and cultivate GM crops more actively. Secondly, farmers’ trust in the government also affects the delayed commercialization of GM crops. The government plays a crucial role in the promotion and application of GM crops, and its stance and policies have significant implications for the commercialization and use of GM crops. If farmers trust and support the government, believing that its decisions and policies are beneficial to them, they will be more willing to accept GM technology and support government policies promoting GM crops. Lastly, age and education levels impact farmers’ acceptance of GM crops, but the effect is not as significant as the core variables mentioned earlier.

Based on these conclusions, this paper offers the following policy recommendations:

Firstly, establishing an efficient government administration system to ensure the public and farmers’ right to know and sense of gain in the commercialization of GM crops is key to addressing the issue of GM crop commercialization. According to the interest identification theory,^45^ political forces can influence the policy-making process by establishing and maintaining interest identification. Therefore, the government should base GM crop commercialization policies on the interests of the public and farmers, considering the accumulation of trust among domestic consumer groups. Otherwise, policy effectiveness may decrease as public consumers’ trust in the government diminishes. The government needs to enhance policy transparency and credibility through open and transparent information disclosure, scientific risk assessment, and effective regulatory measures. This includes periodically releasing information on GM crop research, commercialization progress, and safety monitoring, establishing sound systems and norms so that the public and farmers can understand the development and practical application of the technology.

Secondly, although trust in the government has a significant impact on both consumption and production, it is essential to note that the public perceives different purposes of GM crops differently, implying the need for differentiated policies. At the consumer level, it is crucial to clarify that the public places great importance on whether GM crops are edible, but trust in the government has an even more significant impact. Therefore, high-density interactions between consumers and the government should be promoted to increase trust, advancing policies that use different production targets, safety standards, and safety labels for GM crops with different purposes. Organizing public forums, seminars, and other events that involve experts, scholars, government officials, corporate representatives, and the public and farmers can maximize the effect of differentiated policies for various GM crop purposes. Public consumers can learn about the ingredients, sources, and risk assessments of these GM foods in these interactions, make their own decisions, and continuously require the government and enterprises to manage and control production, sales, and labeling. Simultaneously, the public’s importance placed on the purpose of GM crops indicates that GM food production and sales enterprises need to pay more attention to food safety, strengthen risk assessment, labeling, traceability, and other aspects of GM crops, thereby safeguarding consumers’ rights and interests and improving consumer trust in GM foods.

Next, for farmers, meeting their expectations is the most critical factor. Therefore, compared to other influencing factors, showing farmers the tangible advantages of GM crop products is the path to addressing the delayed commercialization of GM crops in China at the production level. The government needs to adopt effective measures to encourage farmers to accept GM technology, such as providing technical training, information support, and financial policy incentives to help them understand GM crop production methods and market potential and reduce the barriers and risks to technology application.

Finally, education levels also impact the public’s and farmers’ acceptance of GM crops. Therefore, improving the education level of the public and farmers, strengthening GM crop science popularization and publicity, enhancing public scientific literacy and scientific knowledge, reducing public distrust and resistance, and increasing farmers’ awareness and understanding of GM technology and GM crop cultivation techniques remain an essential path to addressing the delayed commercialization of GM crops in China.

## 6. Limitations and Future Directions

This study has certain limitations, such as the sample size, survey period, and geographic region. Future research can further expand the sample scope to enhance the representativeness and generalizability of the study. This study also found some unexpected results, such as the relatively small influence of education level on the public’s and farmers’ acceptance of GM crops, which may be due to factors such as sample bias and cultural factors. To gain a deeper understanding of these unexpected findings, future research can explore other possible explanations and further investigate additional factors that may influence the public’s and farmers’ acceptance of GM crops.^44,45^

## Appendix

**Table:**

**Survey Questionnaire**	
1. Basic Information 1.1 Gender: 1.2 Age: 1.3 Education level: 1.4 Occupation:	
2. Understanding of Genetically Modified (GM) Crops 2.1 Are you familiar with what GM crops are? 2.2 What do you think are the main GM crops cultivated in our country? 2.3 How do you view the safety of GM crops? 2.4 What sources do you use to obtain information about GM crops?	
3. Attitude toward GM Crops 3.1 What do you think is the impact of GM crops on the environment? 3.2 How do you view the economic benefits of GM crops for farmers? 3.3 How do you view the government’s policy inclination toward promoting and regulating GM crops?	
4. Purchasing and Using GM Products Behaviour 4.1 When purchasing food, do you pay attention to whether it is a GM product? 4.2 How often do you purchase GM products? 4.3 What are the main factors you consider when purchasing GM products? 4.4 Are you willing to pay a premium for non-GM products?	
5. Farmers’ Expectations and Needs (for farmer respondents only) 5.1 Have you ever planted GM crops? 5.2 If you have, please describe your experience in planting GM crops. 5.3 What are your needs for training in GM crop cultivation technology? 5.4 What are your needs in terms of policy support? 5.5 What are your needs regarding market information? 5.6 Please describe any other expectations and needs you have in GM crop cultivation.	
Thank you for your participation and support! Please be sure to complete the questionnaire truthfully, as we will use the results to conduct relevant research and provide strong support for the promotion of GM crop commercialization and related policy-making.	
